# Biochemical characterization of the self-sacrificing *p*-aminobenzoate synthase from *Nitrosomonas europaea* reveals key residues involved in selecting a Fe/Fe or Mn/Fe cofactor

**DOI:** 10.1007/s00775-025-02109-w

**Published:** 2025-03-13

**Authors:** Spenser Stone, Logan Peters, Charlotte Fricke, W. Keith Ray, Kylie D. Allen

**Affiliations:** 1 Department of Biochemistry, Virginia Tech, Blacksburg, VA USA; 2 Present Address: Department of Cancer Biology, Vanderbilt University, Nashville, TN USA; 3 Present Address: Department of Forensic Science, Virginia Commonwealth University, Richmond, VA USA

**Keywords:** Metalloenzyme, Metal specificity, Oxygenase, Self-sacrificing enzyme, Folate biosynthesis, *p*-aminobenzoate

## Abstract

A noncanonical route for *p*-aminobenzoate (pABA) biosynthesis in select bacteria utilizes a novel self-sacrificing heme oxygenase-like domain-containing oxidase/oxygenase (HDO) superfamily member. The recently characterized self-sacrificing pABA synthase from *Chlamydia trachomatis* (“CADD”) requires manganese and likely employs a heterobimetallic Mn/Fe cofactor. A conserved active site tyrosine residue is cleaved from the protein backbone to serve as the substrate for pABA synthesis and a lysine residue is the amino group donor. Here, we investigated the orthologous pABA synthase from the ammonia-oxidizing bacterium, *Nitrosomonas europaea*, which we refer to as *Ne*PabS. Consistent with the previously studied *C. trachomatis* enzyme, purified *Ne*PabS produces pABA in vitro in a reaction that only requires a metal cofactor, molecular oxygen, and a reducing agent, but no other substrates. Interestingly, maximal activity was observed with the addition of only iron as opposed to manganese and iron; thus, *Ne*PabS utilizes the more traditional Fe/Fe cofactor employed by most characterized HDO superfamily members. The self-sacrificing residues were confirmed to be Tyr25 and Lys159, which are the corresponding self-sacrificing residues in the CADD reaction. Strikingly, we could switch the metal dependence (Fe/Fe to Mn/Fe) and significantly improve the activity (~ twofold) of *Ne*PabS by substituting two phenylalanine residues with tyrosine residues (F148Y/F177Y), thus rendering the enzyme more similar to CADD. These results demonstrate that these two aromatic residues play an essential role in dictating metal specificity and potentially the proposed radical translocation process that facilitates the tyrosine cleavage reaction for pABA synthesis.

**Graphical abstract:**

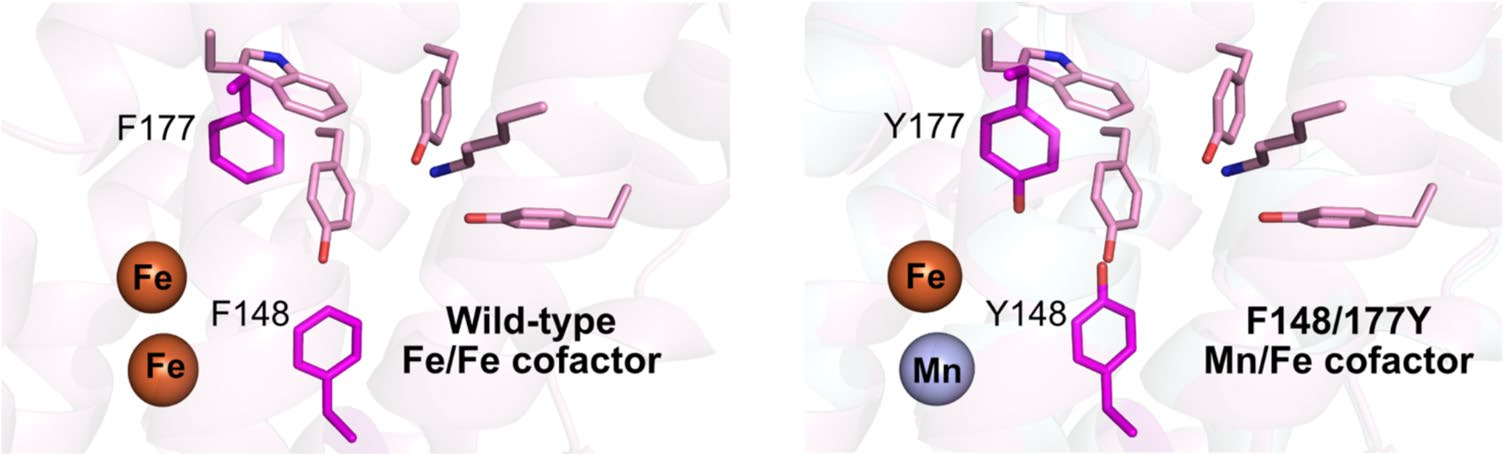

**Supplementary Information:**

The online version contains supplementary material available at 10.1007/s00775-025-02109-w.

## Introduction

**Fig. 1:**
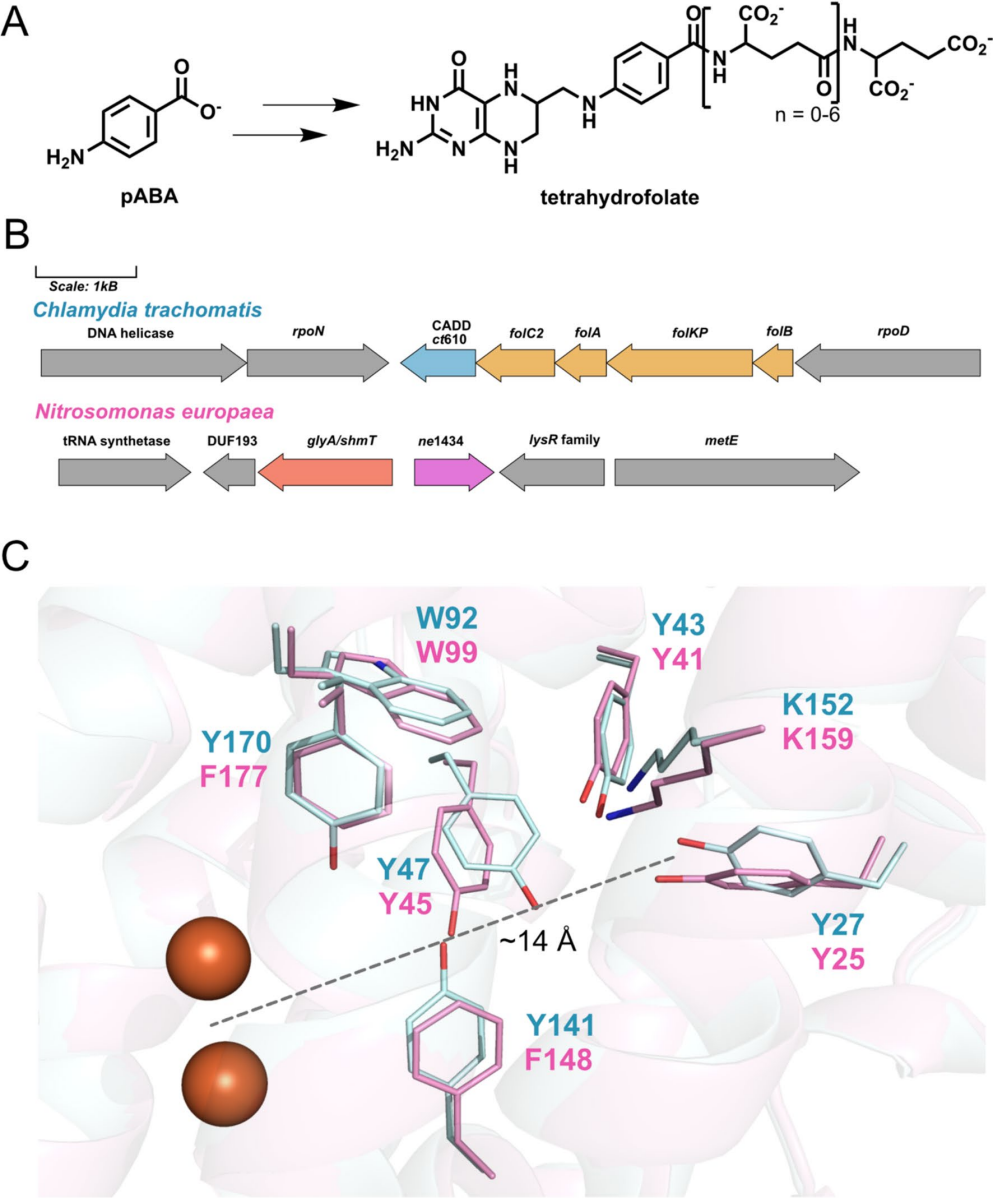
Comparison of pABA synthases from *C. trachomatis* and *N. europaea*. **A** Structure of pABA—required for the de novo biosynthesis of the one-carbon carrier cofactor, tetrahydrofolate. **B** Gene clusters containing the respective pABA synthases in *C. trachomatis* (*ct*610, “CADD”) and *N. europaea* (*ne*1434). **C** Active site of CADD (PDB ID: 1RCW, pale cyan) compared to an AlphaFold 3-predicted structure of *Ne*PabS (pink). The self-sacrificing residues are Y27/25 and K152/159. Other key aromatic residues investigated in this study are also highlighted. The full structure alignment is shown in Figure S3

The heme oxygenase-like domain-containing oxidase/oxygenase (HDO) superfamily comprises an emerging group of non-heme iron enzymes that catalyze diverse reactions including N-oxygenation, oxidative C–C bond cleavage, methine excision, and nitrile formation [1–16]. The first structurally characterized HDO member was the chlamydia protein associated with death domains (CADD) from *Chlamydia trachomatis*, which revealed a seven-helix bundle similar to the heme oxygenase structure*,* but containing a diiron (Fe/Fe) active site coordinated by three histidine residues and three carboxylate residues (Online Resource 1, Fig. S1) [17]. CADD was originally identified as having an important role in inducing host-cell apoptosis during the unique biphasic lifestyle of *C. trachomatis,* an obligate intracellular pathogen [18]; while, more recently, CADD was demonstrated to be involved in a novel pathway for folate biosynthesis [19, 20] (Fig. 1A, B). The enzyme catalyzes a remarkable self-sacrificing reaction to generate *p*-aminobenzoate (pABA), where the side-chain of an active site tyrosine residue (Tyr27) is cleaved from the protein backbone to provide the scaffold for pABA synthesis and the sidechain of a lysine residue (Lys152) is the source of the pABA amine [21–23].

Although CADD was originally crystallized with a Fe/Fe cofactor [17], maximal pABA synthesis in vitro requires manganese and the physiologically relevant pABA synthase form of the enzyme is expected to contain a Mn/Fe cofactor [21, 22]. Indeed, a recent crystal structure obtained with CADD containing Mn/Fe in the metal binding site demonstrated that the heterobimetallic cluster can be accommodated within the HDO scaffold (Online Resource 1, Fig. S1) [24]. Other O_2_-activating enzymes with a Mn/Fe cofactor include select members of the ferritin-like dimetal oxidases—the R2 subunit of class1c-ribonucleotide reductase (RNR) originally characterized in *C. trachomatis* [25] and the R2-like ligand binding oxidase [26]—as well as the recently described amidohydrolase-related dinuclear oxygenases (AROs) [27, 28]. The mechanism for pABA synthesis by CADD remains unresolved, but is thought to involve: (1) molecular oxygen reaction with the reduced Mn^2+^/Fe^2+^ cofactor to produce an activated oxo species that can perform radical initiation and/or oxygen insertion; (2) RNR-like [29] radical translocation through aromatic residues to the substrate tyrosine residue (a distance of about 14 Å, Fig. 1C); (3) radical-based cleavage of the Cα–Cβ bond of Tyr27 to release the aromatic scaffold for pABA synthesis, leaving a glycine residue remaining on the product form of the enzyme; (4) oxidation of the tyrosine-derived substrate via further reactions with O_2_ to generate the carboxylate portion of pABA; (5) reaction with the ε-amino group of lysine (Lys152) to generate the amine of pABA.

An orthologous HDO-family pABA synthase (~ 40% identity and ~ 60% similarity to CADD) has been identified in *Nitrosomonas europaea*—an ammonia-oxidizing soil bacterium. Genetic studies demonstrated that the *ne*1434 gene from *N. europaea* could complement an *E. coli* Δ*pabABC* strain, which would otherwise require pABA or folate supplementation to survive [30]. Unlike CADD, *ne*1434 is not genomically clustered with other folate biosynthesis genes, although it is adjacent to the gene encoding a putative serine hydroxymethyltransferase (*shmT*/*glyA*) that synthesizes methylenetetrahydrofolate from glycine and tetrahydrofolate (Fig. 1B). Given the remarkable and highly unusual self-sacrificing reaction performed by CADD, we set out to biochemically characterize the ortholog in *N. europaea*, which we refer to here as *Ne*PabS. Primary sequence analysis (Online Resource 1, Fig. S2) revealed that the self-sacrificing residues are conserved in *Ne*PabS, but the enzyme contains phenylalanine residues in place of two of the active site tyrosine residues adjacent to the dimetal cofactor in CADD (Fig. 1C). We performed site-directed mutagenesis and proteomics analyses to confirm the essential nature of the self-sacrificing residues as well as the potential significance of the Tyr to Phe substitutions. We demonstrate that *Ne*PabS is a self-sacrificing pABA synthase that utilizes a Fe/Fe cofactor as opposed to a Mn/Fe cofactor. Interestingly, converting the two phenylalanine residues adjacent to the metal cofactor to tyrosine residues switches the metal preference of the enzyme and substantially improves the pABA synthase activity.

## Methods

### Materials

Isopropyl-β-D-thiogalactopyranoside (IPTG), ampicillin, and dithiothreitol (DTT) were acquired from Gold Biotechnology, Inc. Ferrous ammonium sulfate, ascorbic acid,* p*-aminobenzoic acid, and ^18^O_2_ gas were from Millipore-Sigma. Manganese(II) sulfate monohydrate was from Alfa Aesar. LC–MS grade solvents were from Fisher Scientific and primers for mutagenesis were from Integrated DNA technologies (IDT). All other standard reagents and supplies were from Genesee Scientific or Research Products International unless otherwise specified.

### Cloning, overexpression, and purification

The *ne1434* gene (Accession: WP_011112002.1) from *N. europaea*—along with associated overlaps for HiFi Assembly (New England Biolabs)—was obtained as a gBlock from IDT (see sequence in Online Resource 1). The gBlock was assembled with NdeI-digested pet15b using the NEB HiFi Assembly Master Mix according to the manufacturer’s instructions. A portion (2 μl) of the assembly reaction was transformed into Zymo Mix and Go *E. coli* DH5α cells and the purified plasmids from the resulting transformants were sequence verified by Plasmidsaurus.

The resulting plasmid encoding *Ne*PabS with an N-terminal hexahistidine tag was transformed into *E. coli* BL21 and plated on a Luria–Bertani (LB) agar plate supplemented with 100 µg/mL ampicillin. Single colonies were used to inoculate flasks with 50 mL of LB and 100 µg/mL ampicillin, which were incubated overnight at 37 °C and shaking at 300 RPM. The next day, a 15 mL aliquot was used to inoculate two 3 L flask containing 1.5 L of M9 minimal medium with casamino acids (50 mM Na_2_HPO_4_, 20 mM KH_2_PO_4_, 10 mM NaCl, 20 mM NH_4_Cl, 0.5% glucose, 0.1 mM CaCl_2_, 2 mM MgSO_4_, 5 µM Fe(NH_4_)_2_(SO_4_)_2_ and 2 g/L casamino acids) supplemented with 100 µg/mL ampicillin. The cells were grown at 37 °C with shaking at 200 RPM until they reached an OD_600_ of ~ 0.7. Expression of the *Ne*PabS-encoding gene was then induced by adding 0.2 mM IPTG and the cells were cultured for an additional ~ 18 h at 30 °C. The cells were harvested by centrifugation at 8000 × g for 15 min and stored at − 20 °C until purification. The enzyme produced in cells grown in M9 medium supplemented with iron had the highest in vitro pABA synthase activity compared to enzyme produced in M9 lacking iron, LB, or LB supplemented with an iron chelator (1,10-phenanthroline).

The next steps for protein purification were performed under anaerobic conditions in a Coy vinyl anaerobic chamber (97% N_2_/3% H_2_) to ensure that the enzyme/substrate would not react during the lysis and purification procedures (since O_2_ is required for the reaction). The cell pellet (typically ~ 7 g from 1 L of culture) was thawed and resuspended in ~ 20 mL of anaerobic 50 mM sodium phosphate, 300 mM NaCl, and 20 mM imidazole (pH 7.4) (buffer A). Cells were lysed via sonication and the resulting cell lysate was transferred to a 50 mL Oak Ridge tube with a sealing cap (Thermo Fisher Scientific) and centrifuged at 27,000 × g for 50 min. Back in the anaerobic chamber, the supernatant was loaded onto a gravity flow column (1 by 3 cm, ~ 2 mL of resin) of Ni-nitrilotriacetic acid metal affinity resin (Prometheus, Genesee Scientific), equilibrated with buffer A. The column was then washed with 20 mL of buffer A and *Ne*PabS was then eluted from the column by 10 mL of buffer B (50 mM sodium phosphate, 300 mM NaCl, and 250 mM imidazole, pH 7.4) The purified protein was concentrated to 2.5 mL using an Amicon centrifugal concentrator (10-kDa cutoff, 15 mL; MilliporeSigma) sealed with vinyl tape and then exchanged into 20 mM HEPES, pH 7.5 using a PD-10 desalting column (Cytiva Life Sciences). Protein concentrations were determined by the Bradford method with bovine serum albumin as a standard. For the wild-type protein, an average prep from a 7 g pellet yielded ~ 30 mg of protein and all of the variants studied here also had similar yields.

### Enzyme assays and pABA detection by LC–MS

Activity assays were carried out with fresh protein immediately following purification. The protein was removed from the anaerobic chamber and used to prepare reactions (500 µL) containing 150 µM (4.5 mg/mL) protein and 1.5 mM ascorbate in 20 mM HEPES buffer (pH 7.5). In Fe-only and Mn-only reactions, 300 μM ferrous ammonium sulfate (Fe(NH_4_)_2_(SO_4_)_2_) or manganese(II) sulfate (MnSO_4_) were added. For the mixed metal assays, 150 μM of each metal were added to the reaction and the metal stocks were premixed before being added to the assay. For the control reactions lacking protein, samples contained buffer, 1.5 mM ascorbate, and 150 μM of each metal. Reactions were then incubated at 37 °C for 18 h followed by quenching with 2X CH_3_CN and precipitated protein was removed by high-speed centrifugation. The supernatant was transferred to a new tube and concentrated down to 500 µL by vacuum centrifugation before analysis by liquid chromatography-mass spectrometry (LC–MS).

For LC–MS analysis, a Waters Acquity TQD mass spectrometer with a Waters Acquity UPLC equipped with an Acquity Premier HSS T3 column (2.1 × 100 mm, 1.8 µm particle size) was used with solvent A as 0.1% formic acid in water and solvent B as 100% methanol. The LC program consisted of 3 min at 98% A followed by a 10-min linear gradient to 50% B at a flow rate of 0.3 mL/min and the injection volume was 2 µl. The MS method was a multiple reaction-monitoring method (MRM) scanning three pairs: 138.1 and 120.4, 138.1 and 94.4, and 138.1 and 77.3, with a collision energy of 15 V. The source temperature was 150 °C, the desolvation temperature was 500 °C, the desolvation gas flow was 800 L/h, and the cone gas flow was 50 L/h. MassLynx was used for system operation and data processing. A standard curve was generated using known amounts of authentic pABA treated in the same manner as the enzyme reactions.

For ^18^O_2_ incorporation experiments, reactions were prepared in crimp-top Wheaton vials with addition of reducing agent and Fe(NH_4_)_2_(SO_4_)_2_ in the anaerobic chamber. Experimental vials were flushed with ^18^O_2_ while control vials were flushed with ^16^O_2_ from a syringe filled with air. The reactions were processed as described above and the MS method consisted of a general scan in positive mode from 50 to 200 m*/z*.

### Site-directed mutagenesis

*Ne*PabS variants were generated utilizing the Phusion site-directed mutagenesis kit (Thermo Fisher Scientific) following the manufacturer’s instructions. Primers used for mutagenesis are listed in Table S1 in Online Resource 1. Sanger sequencing services at Virginia Tech were used to confirm the desired mutations in each pET15b construct. Each variant was then expressed and purified in the same manner as the wild-type protein.

### LC–MS/MS analysis of *Ne*PabS-derived peptides

Standard enzyme assays described above were performed with wild-type *Ne*PabS and *Ne*PabS-F148/177Y with 150 µM protein. The wild-type enzyme reactions contained 300 µM Fe(NH_4_)_2_(SO_4_)_2_ while the variant reactions contained 150 µM Fe(NH_4_)_2_(SO_4_)_2_ and 150 µM MnSO_4._ Control samples contained the as-purified protein incubated at 37 °C under aerobic conditions alongside the reaction samples. An aliquot of protein (25 µg) was removed from each sample and diluted to 1 µg/µl in 50 mM triethylammonium bicarbonate, pH 8.5. Cysteine residues were reduced and alkylated by adding 4.5 mM dithiothreitol and incubation at 37 °C for 1 h followed by the addition of 10 mM iodoacetamide and incubation at room temperature in the dark for 30 min. Excess iodoacetamide was inactivated by the addition of 10 mM dithiothreitol. The samples were then digested overnight with 0.5 µg of MS grade trypsin (Thermo Fisher Scientific) followed by a tenfold dilution in 2:98 LC–MS grade acetonitrile:water containing 0.1% formic acid. The resulting peptides were analyzed by LC–MS/MS on an Easy-nLC 1200 liquid chromatography system with a µPAC compatible nanospray emitter, an Easy Spray nanospray source and a tribrid Orbitrap Fusion Lumos mass spectrometer (Thermo Fisher Scientific). The system settings were as previously reported [21]. Proteome Discoverer (Thermo Fisher Scientific) was used for quantitation of peptides among the four sample types as well as for generating the peptide identification results (precursor mass tolerance of $\pm 10 \text{ppm}$ and fragment mass tolerance of $\pm 0.1 \text{Da}$). The protease specificity was set to semi-trypsin with carbamylation of Cys residue as a fixed modification and Tyr to Gly, Lys to aminoadipic acid, Phe to Tyr, oxidation of Met, cyclization of N-terminal Gln to pyro-Glu, acetylation of the protein N-terminus, and deamidation of Asn/Gln as variable modifications.

### AlphaFold 3 structural prediction

A structural model of *Ne*PabS was generated using the AlphaFold Server (alphafoldserver.com) powered by AlphaFold 3 [31] with the query sequence from NCBI accession CAD85345. The resulting highest confidence structure had a pTM score of 0.92 and ~ 85% of the protein had pLDDT scores > 90 (high confidence), with the lower confidence values associated with three short disordered regions (Online Resource 1, Fig. S3), including the C-terminal disordered region that was absent in the CADD crystal structure. The structure was further validated using the Swiss Model Structure Assessment web server [32] and was exported to PyMOL version 3.0.3 for visualization and a structural alignment to CADD (PDB: 1RCW).

## Results

### Overproduction and purification of *Ne*PabS

The gene encoding the putative pABA synthase from *N. europaea* (*ne*1434, *Ne*PabS) was inserted into pET15b with an N-terminal hexahistidine tag, followed by transformation and overexpression in *E. coli* BL21. The protein was highly produced in a soluble form and was purified by nickel-affinity chromatography under anaerobic conditions (Online Resource 1, Fig. S4). Metal-analysis of protein purified from cells expressed in M9 medium supplemented with iron and purified anaerobically revealed only ~ 0.05 mol of iron per mol of protein, indicating that the putative metallocofactor is labile and/or is not fully assembled during heterologous expression. In vitro anaerobic reconstitution with excess ferrous iron followed by removal of unbound iron results in ~ 1 mol iron/protein, further suggesting that the expected dimetal form of the enzyme is not stable and potentially that specific assembly factors are required to generate the complete cofactor.

### Determining the activity and metal specificity of *Ne*PabS

**Fig. 2:**
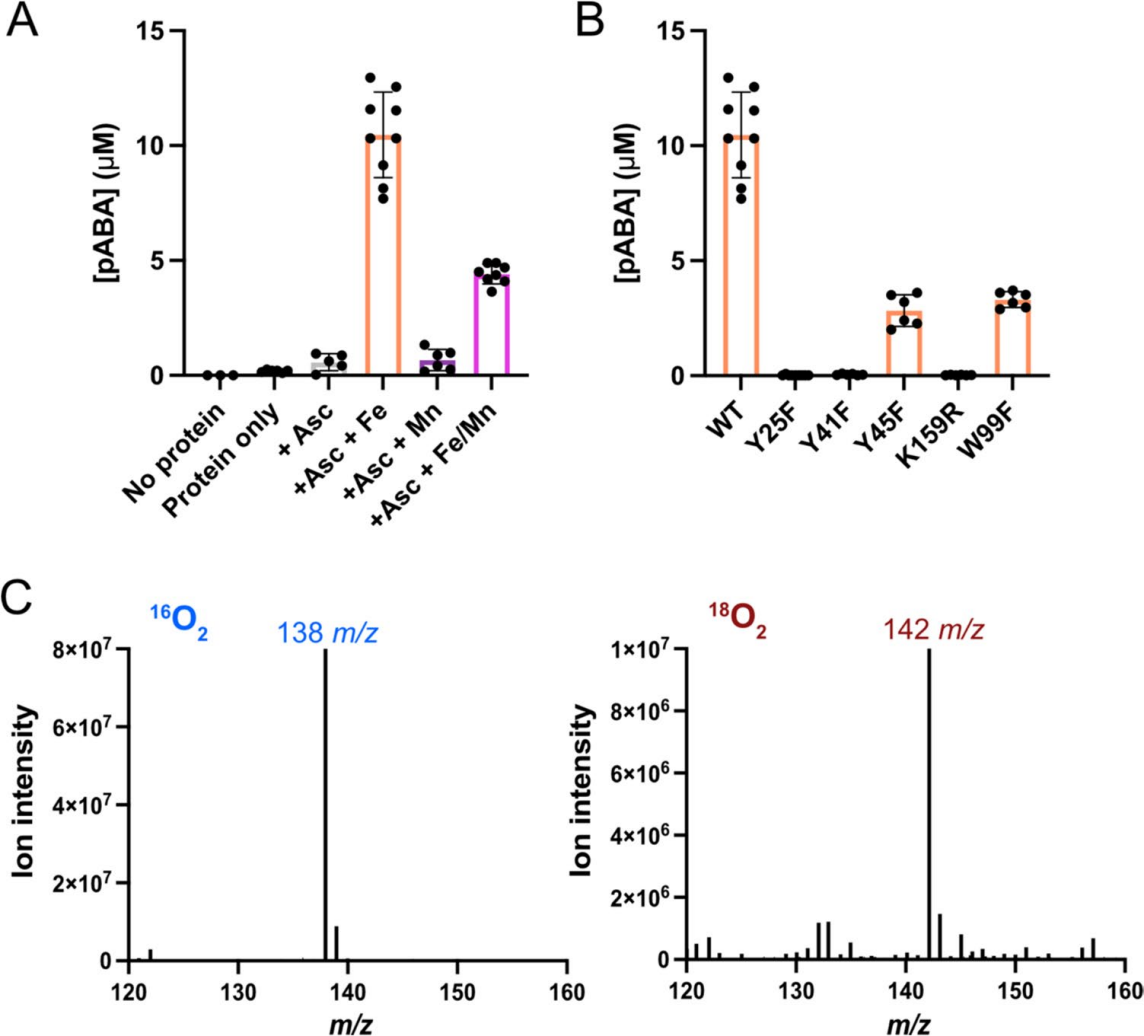
Characterization of pABA synthase activity of *Ne*PabS. **A** Analysis of pABA formation by wild-type *Ne*PabS in different conditions. Samples contained 150 µM protein with 1.5 mM ascorbate and 300 µM ferrous ammonium sulfate for Fe/Fe, 300 µM manganese(II) sulfate for Mn/Mn, or 150 µM of each for Mn/Fe. Data is compiled from reactions using protein from at least two different purifications. **B** Analysis of pABA formation by selected active site variants compared to the wild-type enzyme in the presence of 1.5 mM ascorbate and Fe(II). **C** Incorporation of molecular oxygen into pABA during the *Ne*PabS reaction

With the purified enzyme in hand, we performed assays to assess the pABA synthase activity of the enzyme. The reactions were performed in air under atmospheric O_2_ concentration using ascorbate as the reducing agent and with the addition of either iron, manganese, or both iron and manganese. Although CADD employs a Mn/Fe cofactor, *Ne*PabS exhibited maximal pABA production in reactions containing 2 molar equivalents of iron, which was ~ 2 × higher activity compared to reactions with 1 equiv. of iron and 1 equiv. of manganese (Fig. 2). Thus, *Ne*PabS likely employs the more traditional Fe/Fe cofactor. The nature of the self-sacrificing reaction means that, at most, one molecule of pABA can be produced for every pABA synthase active site. Thus, in reactions that contain 150 μM (monomer concentration) of *Ne*PabS, we would expect 150 μM of pABA produced. However, only ~ 10 μM of pABA is observed in reactions with Fe/Fe, indicating less than 10% of the enzyme is active. Time course experiments revealed that the maximal amount of pABA is produced after ~ 18 h and extended incubation periods do not result in increased pABA production (Online Resource 1, Fig. S5). Additionally, removing the his-tag via protease cleavage before the assays does not result in improved activity. We also confirmed that addition of potential substrates—including chorismate, tyrosine, and *p*-hydroxybenzoate—did not increase the pABA synthase activity observed, further supporting that the enzyme itself is the substrate. Taken together, *Ne*PabS is a pABA synthase, but displays substantially lower in vitro activity compared to CADD, where ~ 30–90% conversion is observed [21, 22].

To gain insight into the importance of conserved aromatic residues in the *Ne*PabS active site, we created, purified, and analyzed the pABA synthase activity of several variants (Fig. 2B). Consistent with our previous results with CADD [20], Tyr25 and Tyr41 along with Lys159 are essential for pABA production by *Ne*PabS (Figs. 1C, 2B). Tyr25 and K159 are the expected sacrificial substrates for pABA synthesis while Tyr41 plays a yet unknown essential role. Based on the mutagenesis results, Tyr45 and Trp99 may facilitate the radical translocation process since converting these to phenylalanine residues significantly decreased pABA production (Fig. 2B).

To assess whether *Ne*PabS incorporates molecular oxygen into the final pABA product, we performed experiments in the presence of ^18^O_2_ compared to air containing ^16^O_2_. *Ne*PabS-derived pABA incubated with ^18^O_2_ displayed a [M + H]^+^ ion at 142 m*/z* compared to the 138 m*/z* molecular ion for pABA obtained from reactions in the presence of air, indicating that two oxygen atoms derived from O_2_ are incorporated into the carboxylate of pABA (Fig. 2C).

### Exploring the roles of divergent aromatic residues in *Ne*PabS compared to CADD

**Fig. 3:**
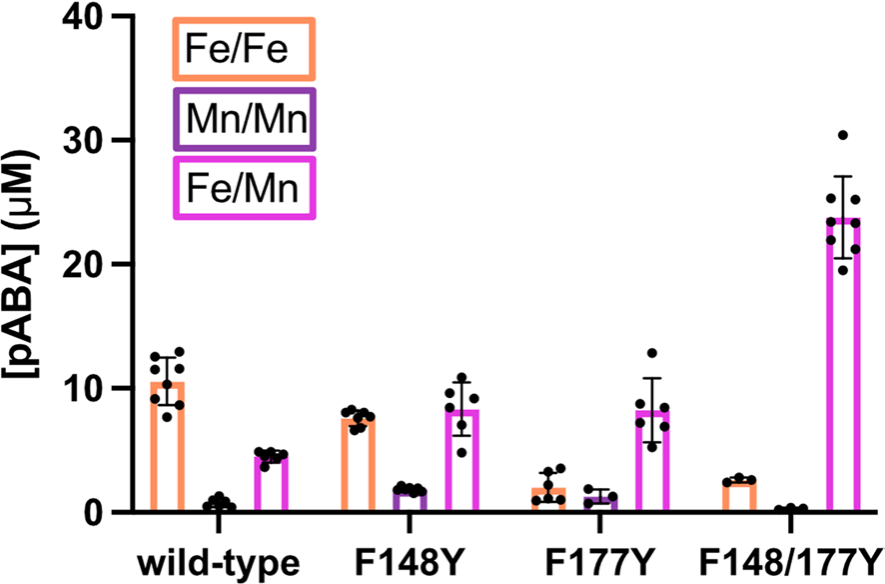
Identification of residues involved in dictating the metal preference of *Ne*PabS. Reactions contained 150 µM protein, 1.5 mM ascorbate, and 300 µM ferrous ammonium sulfate for Fe/Fe, 300 µM manganese(II) sulfate for Mn/Mn, or 150 µM of each for Mn/Fe

The two tyrosine residues adjacent to the dimetal cofactor of CADD—Y170 and Y141 (~ 4 Å and ~ 7 Å from metal site, respectively)—are substituted with phenylalanine residues in *Ne*PabS—F177 and F148 (Figs. S2 and 1C). To determine the potential roles and importance of these residues, we converted them individually and in parallel to tyrosine residues. Interestingly, the F148Y variant resulted in a cambialistic enzyme having similar levels of activity with Fe/Fe compared to Mn/Fe, while the F177Y variant resulted in a clear conversion of the metal preference from Fe/Fe to Mn/Fe (Fig. 3). Converting both residues to tyrosines to generate the double variant—F148/177Y—maintained the strong preference for Mn/Fe and, notably, resulted in ~ twofold higher pABA production compared to the wild-type enzyme and both single variants (Fig. 3). Thus, these two tyrosine residues play a key role in metal cofactor selectivity as well as potentially supporting a putative radical translocation process. It is important to note that the metal content in the as-purified variants was unchanged compared to the wild-type enzyme (Online Resource 1, Table S2), so the differences in metal preference can be confidently attributed to the metals added during the pABA synthase assay.

### Analysis of *Ne*PabS-derived peptides before and after the pABA synthase reaction.

To confirm the identities of the proposed self-sacrificing residues in the *Ne*PabS reaction, we subjected *Ne*PabS samples to trypsin protease cleavage for analysis of the resulting peptides by LC–MS/MS. Based on previous results with CADD, the expected sacrificial tyrosine residue is Tyr25 (Fig. 1C), where the Cα–Cβ bond is cleaved to leave a glycine residue on the product form of the protein [21, 22]. Two major peptides were observed that contain the expected Tyr to Gly substitution at position 25 (Table 1, a complete list of all observed peptides is present in Online Resource 2). One of these (**G**IAWTEGK) is a non-tryptic peptide that is ~ 5× more intense in the reaction compared to the as-purified protein (Table 1). This result suggests that the peptide bond may be cleaved between amino acids 24 and 25 over the course of the putative radical reaction cycle. However, the glycine modification was also observed in the full-length tryptic peptide (HLLQHPF**G**IAWTEGK) in both the as-purified as well as the in vitro reacted protein (Table 1), and we also observed several other non-trypsin-specific peptides in the LC–MS/MS analysis indicating that autoproteolysis occurred on these samples and may not necessarily be related to the pABA synthase reaction.

**Table 1:** Summary of data for major peptides observed with Tyr25 to Gly modification and Lys159 to aminoadipic acid (Aaa) modification

	Major modified peptides	Observed *m/z*	Theoretical *m/z*	Error ppm	Intensity as-purified (× 10^6^)	Intensity after in vitro reaction (× 10^6^)
*Wild-type enzyme*						
	**Tyr25 to Gly peptides**					
	GIAWTEGK, z = 2	431.2295	431.2269	6.0	8.7	43
	HLLQHPFGIAWTEGK, z = 3	578.6456	578.6423	5.7	13	5.7
	**Lys159 to Aaa peptides**					
	PDIAAVAaaIDGLAK, z = 2	663.3736	663.3734	0.3	Not detected	5.8
	SQVPDIAAVAaaIDGLAK, z = 2	820.4542	820.4500	5.1	Not detected	2.4
	ESQVPDIAAVAaaIDGLAK, z = 3	590.3203	590.3109	16	2.8	26
	HAFESQVPDIAAVAaaIDGLAK, z = 2	1062.5580	1062.5531	4.6	0.70	8.2
	AALHAFESQVPDIAAVAaaIDGLAK, z = 3	793.7620	793.7518	12.9	0.37	2.6
*F148/177Y variant*						
	**Tyr25 to Gly peptides**					
	GIAWTEGK, z = 2	431.2293	431.2269	5.6	Not detected	37
	HLLQHPFGIAWTEGK, z = 2	867.4644	867.4598	5.3	6.6	12
	HLLQHPFGIAWTEGK, z = 3	578.6465	578.64230	7.3	1.1	10
	**Lys159 to Aaa peptides**					
	PDIAAVAaaIDGLAK, z = 2	663.3656	663.3734	11.7	Not detected	1.8
	SQVPDIAAVAaaIDGLAK, z = 2	820.4552	820.4500	6.3	0.045	6.6
	SQVPDIAAVAaaIDGLAK, z = 3	547.3056	547.3027	5.3	Not detected	3.4
	ESQVPDIAAVAaaIDGLAK, z = 2	884.9686	884.9758	8.1	Not detected	2.2
	HAYESQVPDIAAVAaaIDGLAK, z = 3	714.0402	714.0367	4.9	Not detected	1.9
	YAGLAALHAYESQVPDIAAVAaaIDGLAK, z = 3	933.8300	933.8235	7.0	Not detected	0.65

**Fig. 4:**
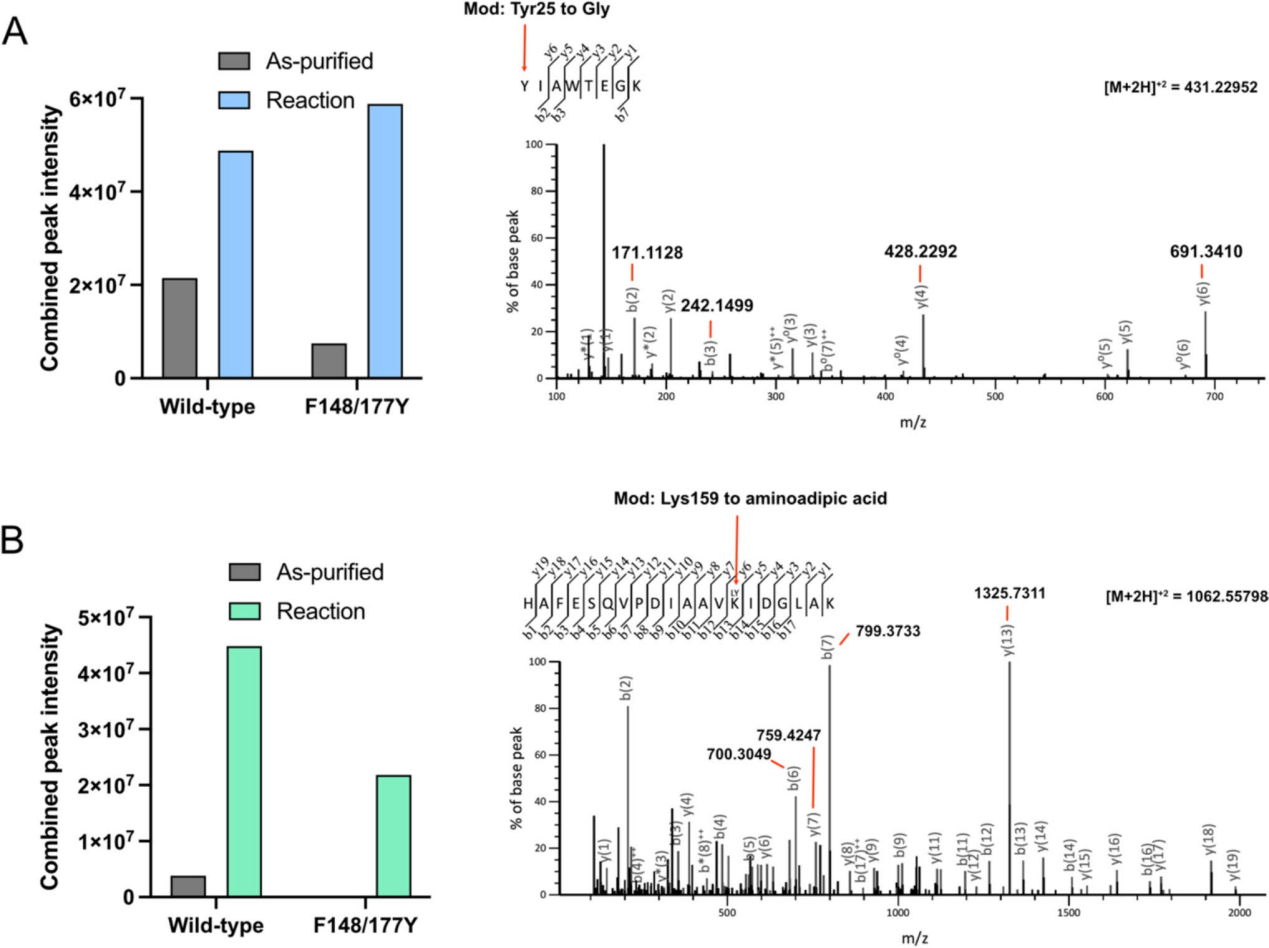
LC–MS/MS analysis of self-sacrificing residues in *Ne*PabS reaction. The bar graphs represent a comparison of combined peak intensities for purified enzyme only compared to in vitro reacted enzyme for peptides containing **A** Y25G modification and **B** K159Aaa modification (intensities for each ion listed in Table 1). The MS/MS spectra for selected peptides with each modification are also shown with key fragment ions labeled, which confirm the identities of the modifications

The combined peak intensities for the two glycine-containing peptides are only ~ 2.2 × more intense in the samples analyzed after the in vitro pABA synthase reaction compared to the samples containing as-purified protein (Fig. 4). This indicates that a portion of the protein has already reacted during the expression in *E. coli* and partially explains our observed low in vitro activity. LC–MS/MS analysis of peptides derived from the *Ne*PabS-F148177Y variant revealed that the two major glycine-containing peptides were ~ 7.4 × more abundant in the reacted protein compared to the as-purified protein, supporting the increased in vitro pABA synthase activity observed with this variant (Fig. 3).

The synthesis of pABA requires an amino group donor, which is an active site lysine residue (Lys152) in the CADD reaction [21, 23], corresponding to Lys159 in *Ne*PabS (Figs. 1C and S2). We observed several abundant peptides containing the expected Lys159 to aminoadipic acid (Aaa) modification (Table 1), where the side-chain amino group is converted to a carboxylic acid. Like the Y25G-modified peptides, the Aaa-containing peptides were more abundant in the reactions compared to the as-purified protein (Table 1 and Fig. 4B), strongly supporting the role of Lys159 as a sacrificial amino group donor during *Ne*PabS catalyzed pABA synthesis. It is important to note that different peptides containing the Lys159 to Aaa modification (as a result of differences in the autoproteolysis locations) were observed in the wild-type protein vs. the F148/177Y variant (Table 1), so the maximal combined intensities cannot be directly compared.

## Discussion

A novel route for pABA biosynthesis involving a self-sacrificing enzyme that uses its own amino acid residues as substrates was originally characterized in the intracellular pathogen *C. trachomatis* [19–22], and bioinformatic analyses indicate that putative self-sacrificing pABA synthases also exist in diverse bacteria including other intracellular pathogens such as *Wolbachia* and *Rickettsia* as well as environmental microbes such as members of *Chloroflexi*, *Sphingomonas*, and *Nitrosomonas*. These pABA synthases are members of the HDO superfamily, which possess a heme oxygenase-like architecture harboring a dinuclear or mononuclear iron-containing cofactor that activates oxygen for oxygenase or oxidase reactions [1, 15]. Interestingly, the only other previously characterized self-sacrificing pABA synthase from *C. trachomatis* is a moonlighting protein that also serves a role in signaling host cell apoptosis [18].

Here, we biochemically characterized *Ne*PabS—the pABA synthase from *N. europaea*, an ammonia-oxidizing soil bacterium. The enzyme catalyzes pABA formation in vitro in the absence of any other substrates besides a redox donor and molecular oxygen, where the two oxygen atoms in the carboxylic acid portion of pABA are derived from molecular oxygen. As opposed to the unique heterodinuclear Mn/Fe cofactor of CADD from *C. trachomatis*, the enzyme from *N. europaea* employs the more traditional Fe/Fe cofactor of the HDO superfamily. The differences in metal requirements in the two pABA synthases likely reflects the need for the intracellular pathogen *C. trachomatis* to minimize iron usage due to host-mediated iron restriction [33], whereas an environmental microbe like *N. europaea* is not expected to encounter strict iron limitation.

Site-directed mutagenesis along with LC–MS based proteomics analysis of *Ne*PabS derived peptides demonstrated that Tyr25 serves as the sacrificial donor of the pABA scaffold while Lys159 is the source of the amino group, consistent with the roles of the corresponding residues in the CADD self-sacrificing reaction [21–23]. *Ne*PabS exhibits low conversion in terms of pABA production in vitro (less than 0.1 pABA/monomer). One explanation for this could be the unstable nature of the metallocofactor as evidenced by the very low incorporation of iron in the as-purified protein (~ 0.05 mol Fe/mol protein) as well as the lack of a complete diiron cofactor after in vitro reconstitution (~ 1 mol Fe/mol protein). Cofactor lability is an established feature of HDO-family members [1] and several HDO enzymes, including BesC, UndA, and AetD, have been termed “substrate-triggered”, where substrate binding facilitates formation of the active cofactor [5, 9, 12, 16]. Thus, it is possible that a small molecule activator is required to bind to the *Ne*PabS active site and allow for productive assembly of the cofactor. This could serve as a regulatory mechanism, so the protein is not wasted when pABA is not needed—especially if the protein has an alternate function in the cell. On the same line of thought, the pABA synthase activity may require allosteric activation and/or complex formation with protein partners at a location outside the active site. Notably, there is a disordered loop consisting of  ~ 10 amino acids that is absent in CADD and may be an interaction site for an activating factor (Online Resource 1, Fig. S3). If only a small portion of the protein is bound to this putative activating factor, it could help explain the low in vitro activity. Additionally, mass spectrometric analysis of *Ne*PabS-derived peptides showed that a portion of the as-purified protein contained the Y25G modification, indicating that some of the enzyme has reacted during expression in *E. coli* and, thus, is unable to generate pABA in vitro. We tried several alternate expression conditions, including excluding iron from the M9 growth medium, but did not see improved in vitro activity.

**Scheme 1:**
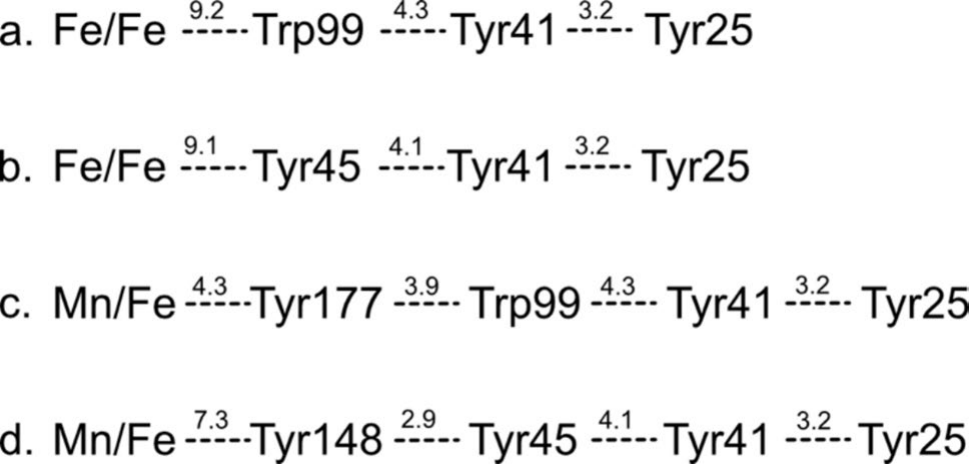
Potential radical transfer pathways in wild-type *Ne*PabS (Fe/Fe-F148-F177) (**a**, **b**) compared to the variant (Mn/Fe-Y148-Y177) (**c**, **d**). Distances are listed in angstroms and are based on the AlphaFold model of *Ne*PabS

Interestingly, pABA production was  ~ twofold higher in the *Ne*PabS F148/177Y variant, in which the two phenylalanine residues adjacent to the metal cofactor are converted to tyrosine residues. This makes the enzyme more similar to CADD, where these residues are already tyrosines in the wild-type enzyme (Fig. 1C and S2). We demonstrated that these aromatic residues are responsible for dictating the metal preference of the enzyme as evidenced by the cambialistic nature of the F148Y variant and the dramatic switch from Fe/Fe to Mn/Fe in the F177Y and F148/177Y variants. Recently, Phan et al. [24] reported that these corresponding residues are also important for metal selectivity in CADD; however, they did not report results for the corresponding double variant that we studied (i.e. Y141/170F in CADD) and they observed creation of a cambialistic enzyme as opposed to a complete conversion of metal dependence that we observed with *Ne*PabS. The increased in vitro pABA production by the F148/177Y variant is likely partially explained by beginning with more of the unreacted substrate form of the purified enzyme. The unique requirement for both manganese and iron maintains more of the inactive form during expression in *E. coli* and, therefore, higher pABA production is observed in vitro once the required metals are supplied to the reaction mixture. This is supported by the lower abundance of Tyr25 to Gly and Lys159 to Aaa modification in the as-purified F148/177Y variant compared to the wild-type enzyme (Fig. 4). These two tyrosine residues adjacent to the metallocofactor may also participate in the proposed radical translocation process—decreasing the distance associated with the electron transfer steps from the activated dimetal cofactor to the substrate tyrosine (Tyr25) (Fig. 1C and Scheme 1), resulting in improved pABA synthase activity.

In summary, we have confirmed the self-sacrificing function of *Ne*PabS in pABA synthesis and demonstrated that the enzyme utilizes a Fe/Fe cofactor that is selected on the basis of two active site phenylalanine residues as opposed to the corresponding tyrosine residues in CADD. Future studies are required to uncover the detailed mechanism of the remarkable self-sacrificing reaction as well as to identify regulatory mechanisms in vivo that are expected to control the pABA synthase activity or direct the protein to alternate functions.

## Supplementary Information

Below is the link to the electronic supplementary material.

Supplementary file1 (PDF 1343 KB)

Supplementary file2 (XLSX 234 KB)

## Data Availability

Data is provided within the manuscript or supplementary information files. Further details are also available from the authors upon request.
