# Supplementary material for: Biochemical characterization of the self-sacrificing p-aminobenzoate synthase from Nitrosomonas europaea reveals key residues involved in selecting a Fe/Fe or Mn/Fe cofactor
Source: J Biol Inorg Chem. 2025 Mar 13;30(3):271–81. doi: 10.1007/s00775-025-02109-w (PMC11965152; doi:10.1007/s00775-025-02109-w)
Supplement: Supplementary file 1 — Supplementary file1 (PDF 1343 KB) [file 775_2025_2109_MOESM1_ESM.pdf]

Supplementary Material (Online Resource 1) for:

Biochemical characterization of the self-sacrificing *p*-aminobenzoate synthase from *Nitrosomonas europaea* reveals key residues involved in selecting a Fe/Fe or Fe/Mn cofactor

Journal of Biological Inorganic Chemistry

Spenser Stone[†], Logan Peters[‡], Charlotte Fricke, W. Keith Ray, and Kylie D. Allen*

Department of Biochemistry, Virginia Tech, Blacksburg, VA 24061

*Address correspondence to: Kylie Allen, kdallen@vt.edu

[†]Current Address: Department of Cancer Biology, Vanderbilt University, Nashville, TN, USA

[‡]Current Address: Department of Forensic Science, Virginia Commonwealth University, Richmond, VA, USA

Supplementary Materials and Methods

Inserting *ne*1434 gene into pET15b. The *ne*1434 gene sequence (WP_011112002.1) with pET15b overlaps for Gibson Assembly [1] in red is shown below. This sequence was purchased as a gBlock from Integrated DNA Technologies and assembled into NdeI-digested pET15b using HiFi Assembly Master Mix (New England Biolabs).

GTGCCGCGCGGCAGCCATATGGCTACAAACACATTCAAGCAACAGGTCGATTCCAT
CATTCAAAGCAGACACCTGCTGCAGCATCCGTTCTACATTGCCTGGACCGAAGGCAA
ACTGACTCGTGAACAGTTACGCCACTATGCTGAGCAGTATTTCTACAATGTGCTGGC
AGAGCCTACCTATCTCAGCGCAGTTCACTTCAATACCCCGCATTTCCATAATGTGGA
AAATAGTGGTGATATCAGCATTCGTCAGGAAGTTCTGAAAAACCTGATCGATGAAG
AACACGGAGAGAAAAATCATCCTGCTCTGTGGAAAGCATTTGCATTTGCGCTGGGA
GCCGACGATGCCAGCCTGACACAAGCCGATGCGCTGCCGGAAACAGAAAACCTGGT
GGCAACTTTCCGTGATATCTGCATCAACGAACCATTCTATGCAGGATTGGCCGCATT
ACATGCGTTCGAATCTCAGGTGCCTGATATTGCTGCCGTCAAAATCGATGGTCTGGC
CAAGTTTTACGGTATGAAAGATCCGGACAGCTACGAATTCTTCTCGGTTCATCAGAC
AGCCGATATCTTCCATTCACAAGCCGAATGGGCAATCATCGAGAAATTTGCCGATAC
ACCGGAAAAACAGGCTGAAGTACTGGCCGCTACACGCCGGGCTTGCGATGCACTGT
GGAAATTCCTGGATGGTATCCACGAAAATTACTGTGCCAATCTCATTTGCGAGGAAA
AAACCGCAGCCACCCTGCACTGATGCTCGAGGATCCGGCTGC

Metal analysis. Quantitation of metals bound to purified *Ne*PabS and its variants was carried out using a Thermo Electron iCAP-RQ inductively coupled plasma mass spectrometer (ICP-MS) according to standard method 3125-B (APHA, AWWA, and WEF, 1998) [2]. Samples and calibration standards were prepared in a matrix of 2% nitric acid by volume.

Iron reconstitution. *In vitro* reconstitution of wild-type *Ne*PabS was performed under anaerobic conditions to attempt to incorporate a stably bound diiron cofactor. To a 2.5 mL sample of purified anaerobic *Ne*PabS (~7 mg/mL) was added 5 molar equivalents of $Fe(NH_4)_2(SO_4)_2$. The sample was gently stirred in the anaerobic chamber for 2 hours, and then exchanged into anaerobic 20 mM HEPES, 7.5 using a PD-10 desalting column (Cytiva Life Sciences).

Tables

Table S1: List of primers used to generate *Ne*PabS variants. Mutations are highlighted in red.

Variant	Forward and reverse primers (5'-3')
Y25F	F: CAGCATCCGTTCTTCATTGCCTGGACC<br>R: GCAGCAGGTGTCTGCTTTGAATGATGGAAT
Y41F	F: CAGTTACGCCACTTCGCTGAGCAGTAT<br>R: TTCACGAGTCAGTTTGCCTTCGGTCCAGGC
Y45F	F: TATGCTGAGCAGTTCTTCTACAATGTG<br>R: GTGGCGTAACTGTTCACGAGTCAGTTTGCC
K159R	F: ATTGCTGCCGTCAGAATCGATGGTCTG<br>R: ATCAGGCACCTGAGATTCGAACGCATGTAA
F148Y and F148Y/F177Y	F: GCATTACATGCGTACGAATCTCAGGTG<br>R: GGCCAATCCTGCATAGAATGGTTCGTTGAT
F177Y	F: CCGGACAGCTACGAATACTTCTCGGTTCATCAG<br>R: ATCTTTCATACCGTAAAACTTGGCCAGACC
W99F	F: ATCATCCTGCTCTGTTCAAAGCATTTGC<br>R: TTTCTCTCCGTGTTCTTCATCGATC

Table S2: Iron content of *Ne*PabS and selected variants determined by ICP-MS.

**Sample**	**mol Fe/mol protein**
wild-type as-purified	0.05
wild-type reconstituted	1.1
F148Y	0.02
F177Y	0.02
F148/177Y	0.05

*All proteins were expressed in M9 medium supplemented with 5 µM $Fe(NH_4)_2(SO_4)_2$ and purified under anaerobic conditions. All samples were also analyzed for manganese, but none was observed above the baseline level in the buffer control.

Figures

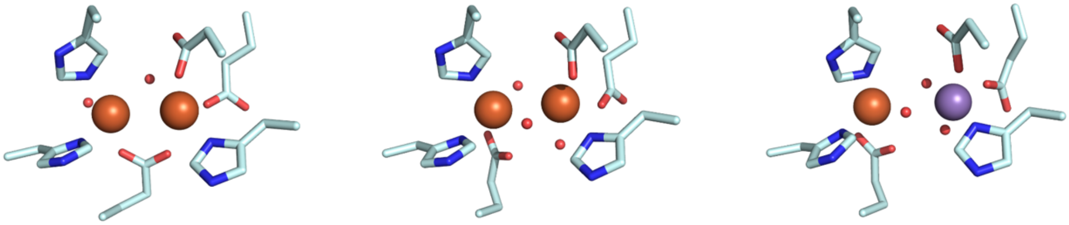

1RCW 8VAB 8VA9

Figure S1. Coordination of the dimetal cofactors in different CADD crystal structures [3, 4]. 1RCW and 8VAB contain two Fe, while 8VA9 contains one Mn and one Fe. Water molecules are shown as red spheres.

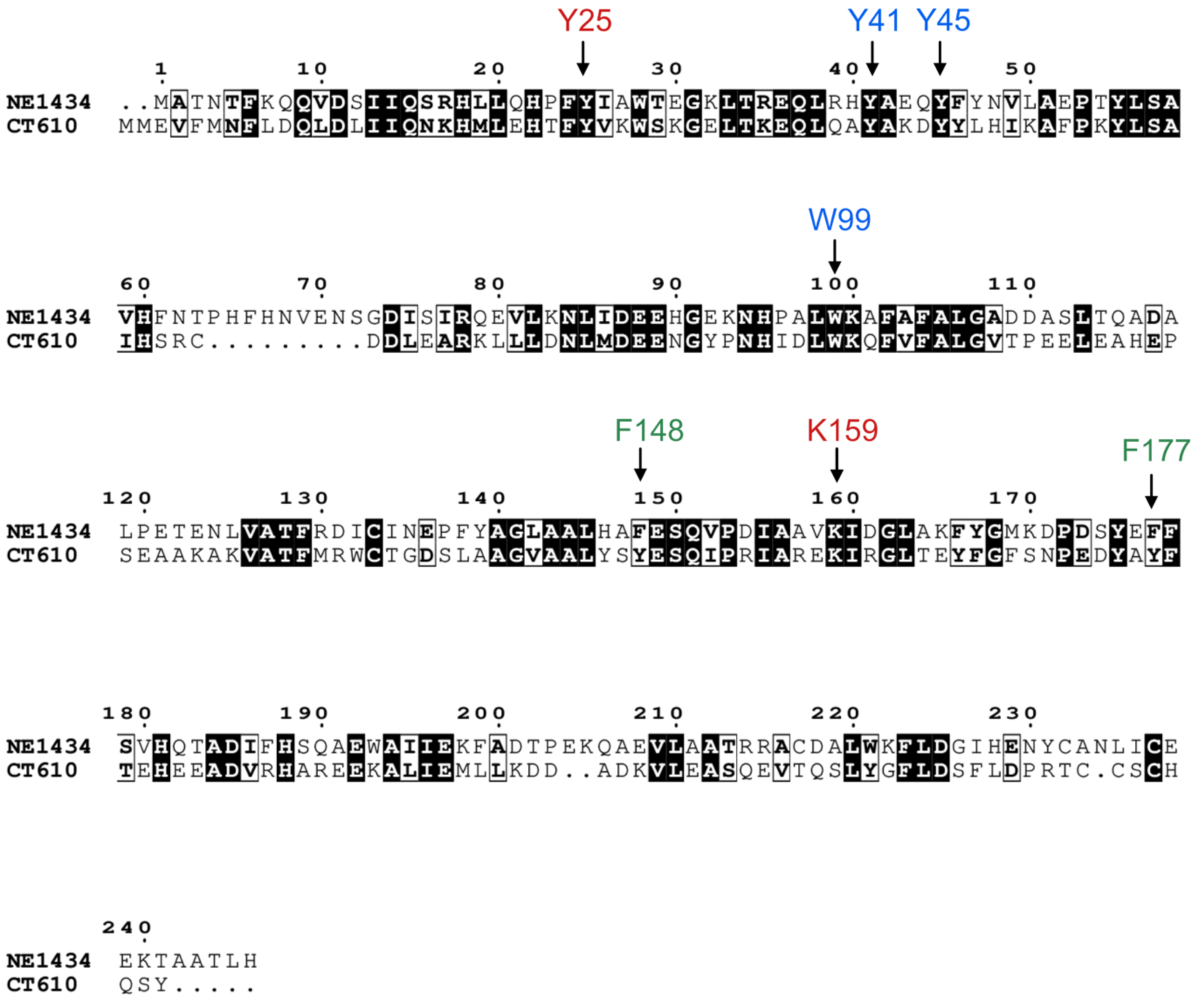

Figure S2. Sequence alignment of *Ne*PabS (NE1434, NCBI accession CAD85345) and CADD(CT610, NCBI accession CAP04311). Conserved self-sacrificing residues are highlighted in red, conserved tyrosine residues and tryptophan in blue, and active site phenylalanine residues in *Ne*PabS that are tyrosine residues in CADD are highlighted in green. Residue numbering is for *Ne*PabS. The sequence alignment was performed in Clustal Omega [5] and visualized with ESPript [6].

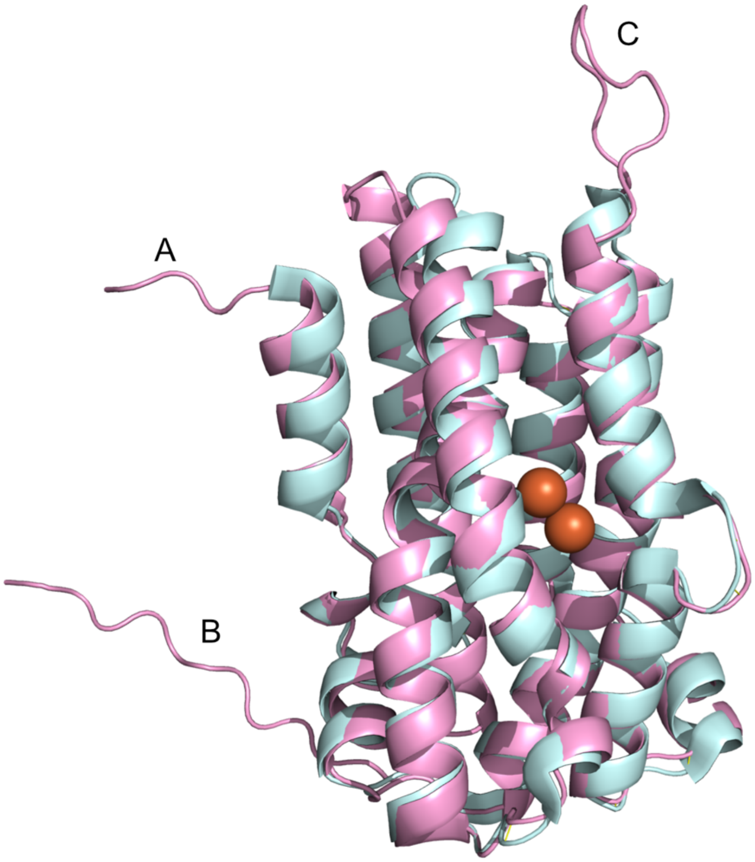

Figure S3. Structural alignment of CADD (PDB ID: 1RCW, pale cyan) with AlphaFold3 model of *Ne*PabS (pink). (A) Disordered N-terminal region consisting of ~5 residues that is absent in CADD with proteomics-predicted start codon [7]. (B) Disordered C-terminal region consisting of ~10 residues. CADD also contains a similar disordered region that was absent in crystal structure [3]. (C) A disordered loop consisting of ~10 residues that are absent in CADD (see sequence alignment above, residues 64-74).

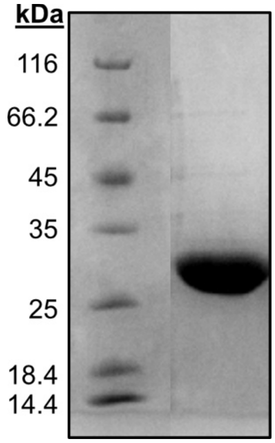

Figure S4. Representative SDS-PAGE gel stained with Coomassie blue showing purified wild-type *Ne*PabS. Each of the variants were obtained in similar purity and yields.

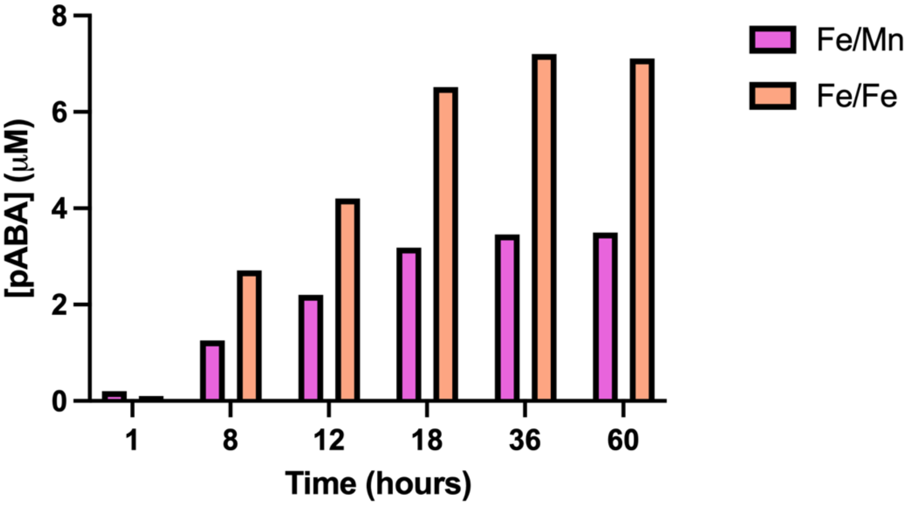

Figure S5. Analysis of pABA synthesis by *Ne*PabS over time. Maximal pABA formation is observed by about 18 hours, which was chosen as the standard reaction time for other experiments reported in this study. The reaction contained 150 μM protein, 1.5 mM ascorbic acid and 2 equiv. of Fe or 1 equiv. of Fe and 1 equiv. of Mn. The amount of pABA formed in this sample was moderately less than that observed in other samples because protein was combined from several preparations that was stored at -20 °C before the time course reaction, as opposed to using freshly purified protein. n=1 for this experiment, so no error bars are shown.

References

1. Gibson, D. G., Young, L., Chuang, R. Y., Venter, J. C., Hutchison, C. A., 3rd & Smith, H. O. (2009) Enzymatic assembly of DNA molecules up to several hundred kilobases, *Nat Methods.* **6**, 343-5.
2. APHA, A., and WEF (American Public Health Association, American Water Works Association, and Water Environment Federation) (1998) *Standard Methods for Examination of Water and Wastewater*, 20th ed. edn, Washington, D.C.
3. Schwarzenbacher, R., Stenner-Liewen, F., Liewen, H., Robinson, H., Yuan, H., Bossy-Wetzel, E., Reed, J. C. & Liddington, R. C. (2004) Structure of the Chlamydia protein CADD reveals a redox enzyme that modulates host cell apoptosis, *J Biol Chem.* **279**, 29320-4.
4. Phan, H. N., Swartz, P. D., Gangopadhyay, M., Guo, Y., Smirnov, A. I. & Makris, T. M. (2024) Assembly of a Heterobimetallic Fe/Mn Cofactor in the para-Aminobenzoate Synthase Chlamydia Protein Associating with Death Domains (CADD) Initiates Long-Range Radical Hole-Hopping, *Biochemistry.* **63**, 3020-3029.
5. Sievers, F., Wilm, A., Dineen, D., Gibson, T. J., Karplus, K., Li, W., Lopez, R., McWilliam, H., Remmert, M., Soding, J., Thompson, J. D. & Higgins, D. G. (2011) Fast, scalable generation of high-quality protein multiple sequence alignments using Clustal Omega, *Mol Syst Biol.* **7**, 539.
6. Robert, X. & Gouet, P. (2014) Deciphering key features in protein structures with the new ENDscript server, *Nucleic Acids Res.* **42**, W320-4.
7. Adams, N. E., Thiaville, J. J., Proestos, J., Juarez-Vazquez, A. L., McCoy, A. J., Barona-Gomez, F., Iwata-Reuyl, D., de Crecy-Lagard, V. & Maurelli, A. T. (2014) Promiscuous and adaptable enzymes fill "holes" in the tetrahydrofolate pathway in Chlamydia species, *MBio.* **5**, e01378-14.
